# Maternal pre-pregnancy obesity affects the uncinate fasciculus white matter tract in preterm infants

**DOI:** 10.3389/fped.2023.1225960

**Published:** 2023-11-15

**Authors:** Joo Young Lee, Hyun Ju Lee, Yong Hun Jang, Hyuna Kim, Kiho Im, Seung Yang, Jeong-Kyu Hoh, Ja-Hye Ahn

**Affiliations:** ^1^ Department of Translational Medicine, Hanyang University Graduate School of Biomedical Science and Engineering, Seoul, Republic of Korea; ^2^ Department of Pediatrics, Hanyang University College of Medicine, Seoul, Republic of Korea; ^3^ Division of Neonatology and Development Medicine, Hanyang University Hospital, Seoul, Republic of Korea; ^4^ Fetal Neonatal Neuroimaging and Developmental Science Center, Boston Children's Hospital and Harvard Medical School, Boston, MA, United States; ^5^ Division of Newborn Medicine, Boston Children's Hospital and Harvard Medical School, Boston, MA, United States; ^6^ Department of Pediatrics, Hanyang University Hospital, Seoul, Republic of Korea; ^7^ Department of Obstetrics and Gynecology, Hanyang University College of Medicine, Seoul, Republic of Korea; ^8^ Department of Obstetrics and Gynecology, Hanyang University Hospital, Seoul, Republic of Korea

**Keywords:** maternal obesity, obesity, pre-pregnancy, preterm birth, diffusion tensor imaging, white matter, uncinate fasciculus

## Abstract

**Background:**

A growing body of evidence suggests an association between a higher maternal pre-pregnancy body mass index (BMI) and adverse long-term neurodevelopmental outcomes for their offspring. Despite recent attention to the effects of maternal obesity on fetal and neonatal brain development, changes in the brain microstructure of preterm infants born to mothers with pre-pregnancy obesity are still not well understood. This study aimed to detect the changes in the brain microstructure of obese mothers in pre-pregnancy and their offspring born as preterm infants using diffusion tensor imaging (DTI).

**Methods:**

A total of 32 preterm infants (born to 16 mothers with normal BMI and 16 mothers with a high BMI) at <32 weeks of gestation without brain injury underwent brain magnetic resonance imaging at term-equivalent age (TEA). The BMI of all pregnant women was measured within approximately 12 weeks before pregnancy or the first 2 weeks of gestation. We analyzed the brain volume using a morphologically adaptive neonatal tissue segmentation toolbox and calculated the major white matter (WM) tracts using probabilistic maps of the Johns Hopkins University neonatal atlas. We investigated the differences in brain volume and WM microstructure between preterm infants of mothers with normal and high BMI. The DTI parameters were compared among groups using analysis of covariance adjusted for postmenstrual age at scan and multiple comparisons.

**Results:**

Preterm infants born to mothers with a high BMI showed significantly increased cortical gray matter volume (*p *= 0.001) and decreased WM volume (*p *= 0.003) after controlling for postmenstrual age and multiple comparisons. We found a significantly lower axial diffusivity in the uncinate fasciculus (UNC) in mothers with high BMI than that in mothers with normal BMI (1.690 ± 0.066 vs. 1.762 ± 0.101, respectively; *p *= 0.005).

**Conclusion:**

Our study is the first to demonstrate that maternal obesity impacts perinatal brain development patterns in preterm infants at TEA, even in the absence of apparent brain injury. These findings provide evidence for the detrimental effects of maternal obesity on brain developmental trajectories in offspring and suggest potential neurodevelopmental outcomes based on an altered UNC WM microstructure, which is known to be critical for language and social–emotional functions.

## Introduction

As the prevalence of obesity has steadily increased, the prevalence of obesity among women of childbearing age has also increased, with rates of 65.8% and 45.5% in the United States and the Republic of Korea, respectively (1, 2). In particular, during the COVID-19 pandemic, maternal pre-pregnancy body mass index (BMI) and obesity rates increased with steeper slopes than those before the COVID-19 era (3). Although epigenetic changes, in addition to genetic factors, may play a role in the interactions between maternal and offspring obesity, the increasing prevalence of maternal obesity has become a prominent health concern worldwide. Maternal obesity leads to several complications, such as gestational hypertension, pre-eclampsia, gestational diabetes mellitus, and delivery of preterm birth, which are important predictors that negatively affect fetal, infant, and childhood outcomes (4–7).

Several studies have focused on maternal obesity-associated programming of offspring metabolism and childhood obesity (8–11). In addition to offspring obesity, a growing body of evidence from epidemiological studies, systematic studies, and meta-analyses suggests that maternal obesity is associated with adverse neurodevelopmental outcomes and psychiatric disorders in the offspring from infancy to childhood (12–15). A large prospective study showed an inverse relationship between maternal pre-pregnancy BMI and cognitive scores of children at age 7 years (16). A meta-analysis documented that mothers with overweight/obese pre-pregnancy have an increased incidence of attention deficit hyperactivity disorder and autism spectrum disorder in their offspring during childhood (17, 18). These findings support the mechanisms underlying the transgenerational effects of maternal obesity on the developing brain during extremely dynamic periods.

However, determining the confounding variables present from infancy to childhood is difficult, which indicates that maternal obesity directly affects offspring neurodevelopment during childhood. Recent advanced magnetic resonance image (MRI) neuroimaging techniques have significantly improved our understanding of the early brain development of offspring born to obese mothers by detecting subtle changes in the brain and providing comprehensive characterizations (19). Investigation of MR neuroimaging of offspring at the earliest possible time can provide early evidence of the effects of maternal obesity on offspring neurodevelopment. To the best of our knowledge, only seven studies have investigated the altered structural and functional brain development in neonates associated with maternal obesity using neuroimaging (Supplementary Table S1) (20–26). In a functional MRI study, four functional domains associated with adult obesity (viz., sensory processing, reward processing, cognitive control, and motor control) showed altered functional connectivity even in the offspring of mothers with overweight and obesity (23). The functional connectivity of the left superior frontal gyrus (24) and left thalamus (25) is principally increased in the offspring of obese mothers and is positively associated with maternal pre-pregnancy BMI. In contrast, the dorsal anterior cingulate in the prefrontal cortex shows a lower functional connectivity, which is negatively correlated with pre-pregnancy BMI (22). In particular, diffusion tensor imaging (DTI), which has the advantage of using isotropic diffusion to visualize axon bundles, provides information about the white matter (WM) microstructural integrity by non-invasively analyzing the orientation of the diffusion of water molecules within brain tissues (27). Ou et al. (21) showed that the fractional anisotropy (FA) value of widespread frontal WM in offspring from maternal obesity was significantly lower than that in newborns born from mothers with normal BMI and was negatively correlated with maternal adiposity.

Preterm birth itself increases the risk of neurodevelopmental morbidity and long-term cognitive disabilities. Immature synaptic formation and axonal growth disrupt the structural and functional development of the cortex and WM, even in the absence of focal brain lesions (28). During this critical period, premature births are likely to exhibit different brain developmental patterns because they are exposed to many stresses in the extrauterine environment and intrauterine stress. Current evidence suggests that pre-pregnancy obesity may be more detrimental to the fetus than weight gain during pregnancy (29). Although mechanically still defined, metabolic changes due to maternal pre-pregnancy obesity affect the expression of epigenetic markers in the fetus, thus influencing the epigenetic programming during embryogenesis. In addition, a substantial Korean Pregnancy Outcome Study, which examined the impacts of maternal pre-pregnancy BMI and weight gain during pregnancy, reported that high pre-pregnancy BMI increase the risk of obstetric complications during the perinatal period by more than 2.5 times (30). Of note, an inverse relationship has been observed between excessive weight gain during pregnancy and the incidence of preterm birth. Hu et al. (31) reported that excessive gestational weight gain (GWG) significantly reduces the risk of preterm birth compared with adequate GWG, whereas low GWG increases the risk of preterm birth. These findings indicate that maternal pre-pregnancy obesity has more potential adverse effects on the offspring than weight gain during pregnancy. The question of when or how dysregulated signals from mothers with obesity reach the developing brain is very complex and our understanding is still limited by inconclusive evidence. Recent advances in neuroscience point to the placenta as a critical organ that may transduce the response to stress, immune function, and oxidative stress caused by maternal obesity; the effect of maternal obesity on the placenta may prime the fetal brain in early or pre-pregnancy for later psychiatric disorders (32). The prenatal influence of maternal obesity on neurocognitive development in offspring should be elucidated in preterm infants at the earliest time after birth to minimize the adverse effects associated with extrauterine life. In a fetal cohort study between the gestational age (GA) of 26 and 39 weeks, Norr et al. (33) demonstrated that the functional connectivity increased in the left inferior frontal gyrus of the within hemisphere, whereas it decreased in the anterior prefrontal cortical regions of the cross-hemisphere in the offspring with maternal obesity. Reynolds et al. (26) reported adverse long-term outcomes of preterm infants born to mothers with obesity, in which pre-pregnancy maternal obesity was associated with lower language scores at 2 years of age and an increased risk of positive screening for autism.

Although research on the effect of maternal obesity in pregnancy on full-term neonates as a prenatal factor is being actively conducted, unfortunately, neurodevelopmental studies on premature neonates born to mothers with obesity during pre-pregnancy are still limited. Preterm infants who are at a high risk of disrupted brain development at even near-term age have the advantage of obtaining opportunities for early intervention and providing information on vulnerability to maternal obesity. However, the effect of maternal obesity on WM development in preterm infants without apparent brain injury has not been investigated using MRI and DTI. We hypothesize that pre-pregnancy maternal obesity mediates brain volume and WM microstructural development at term-equivalent age (TEA) in preterm infants.

## Methods

### Maternal BMI and weight gain during pregnancy

The BMI classifications for obesity by the World Health Organization (WHO) and the National Institutes of Health (NIH) for White, Hispanic, and Black individuals were adopted. However, the risk associated with maternal obesity might be underestimated when these classifications are applied to the Asian population. The WHO and NIH guidelines for Asian individuals define overweight as a BMI of 23–24.9 kg/m^2^ and obesity as a BMI of >25 kg/m^2^ (34). Pre-pregnancy BMI was classified as normal (BMI = 18.5–24.9 kg/m^2^) and obese (BMI > 25 kg/m^2^). The maternal pre-pregnancy weight was measured at the first antenatal visit and within approximately 12 weeks before pregnancy. Data on maternal height and pre-pregnancy weight were collected from the medical records. The maternal pre-pregnancy BMI was calculated as follows: weight (kg)/height (m)^2^. The total GWG was calculated as the difference between the weight at delivery and maternal pre-pregnancy weight. Maternal weight gain during pregnancy was adjusted for the length of gestation. It was calculated by dividing the pregnancy weight gain by the total weeks of gestation for a measure of kilograms per week.

### Subjects

This study was part of a prospective observational cohort study on brain development and outcomes in premature infants conducted at the Hanyang Inclusive Clinic for Developmental Disorders at the Hanyang University College of Medicine between 2018 and 2021. Approval for this protocol and scanning procedures of this study was granted by the Institutional Review Board of the Hanyang University Hospital, and informed consent was obtained from the parents of all infants included in this study. The inclusion criteria for preterm infants were as follows: birth at very preterm infants under 32 weeks of gestation without major congenital malformations, no evidence of intraventricular or intracranial hemorrhage greater than grade I, no evidence of intrauterine growth retardation, and availability of MRI at near-term age between 36 and 41 weeks of postmenstrual age (PMA). The exclusion criteria were as follows: infants born to mothers with pregnancy complications, such as pre-eclampsia and type 2 diabetes, medications during pregnancy known to influence fetal growth, smoking or alcohol drinking, sexually transmitted diseases, and medical conditions, such as seizure disorders and serious psychiatric disorders. In addition, neonates born preterm infants with medical conditions, such as congenital malformations, or those unable to complete a brain MRI examination during natural sleep were also excluded. We included 32 infants who had a calculable maternal BMI at the first prenatal visit and were born at the neonatal intensive care unit of the Hanyang University Seoul Hospital. A total of 16 preterm infants of mothers with a high BMI [(GA): 28.36 ± 1.62 (mean ± standard deviation), range: 26.0–31.6; sex: 5/11 (male/female)] were included in this study. Chorioamnionitis was defined as histologic chorioamnionitis or umbilical cord vasculitis of grade 2 or greater, according to the grading system suggested by Salafia et al. (35). In total, 16 control preterm infants of mothers with normal-BMI [GA: 28.38 ± 1.83, range: 25.6–32.0; sex: 6/10 (male/female)] were matched for GA and sex. All demographic and clinical data including maternal information, GA, birth weight, sex, head circumference, Apgar score, and neonatal clinical factors were prospectively recorded.

### MRI data acquisition

MRI scans of preterm infants were performed during natural sleep at TEA using a 3.0-T MRI scanner (Philips Real-Time Compact Magnet 3.0-T MRI System; Achieva 3.0 T X-Series) equipped with a 16-channel SENSE head coil. T1- and T2-weighted images were obtained using sagittal and axial T1 spin-echo sequences [400/25/2, repetition time (TR)/echo time (ms)/signal intensity average] and axial turbo spin-echo sequences (3,000/100/1), respectively. During image acquisition, cushions were placed between the radiofrequency coil and the subject. DTI was performed using a single-shot spin-echo-planar sequence with a SENSE factor of 2 and an echo-planar imaging factor of 51. The acquisition parameters were as follows: TR/TE, 8,100/75 ms; matrix size, 112 × 112; field of view, 224 mm; 74 axial sections. The slice orientation was axial with a 2.0-mm thickness and parallel to the anterior-posterior commissure line, and 32 directions using an electrostatic gradient model (*b* = 800) were used for diffusivity measurement. All subjects were fed well before the scan, and sedative medications were not used.

### Brain volumetric analysis

**Figure 1:**
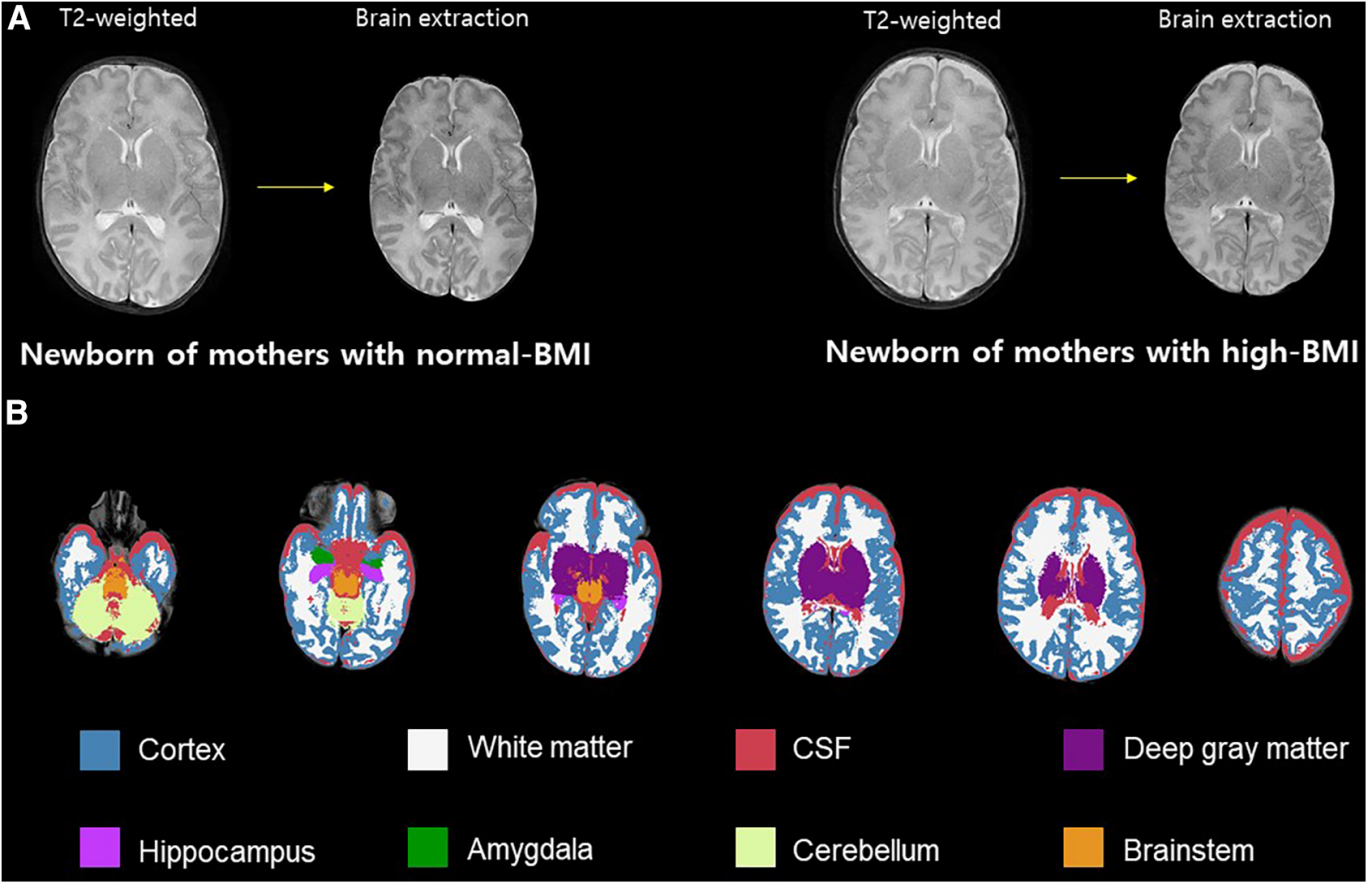
Volumetric analysis. Brain skull excluded from T2-weighted image (**A**). Example axial slices overlaid on T2-weighted images and segmented into eight brain morphological tissues (**B**). BMI, body mass index; CSF, cerebrospinal fluid.

To compare brain volume measurements in the two groups, we used an advanced segmentation technique, the Morphologically Adaptive Neonatal Tissue Segmentation (MANTiS) toolbox (36). This pipeline is an extension of unified segmentation in the Statistical Parametric Mapping (SPM) software, which classifies T2-weighted MR images of the brain into the following eight brain regions: cortical gray matter (GM), WM, deep GM, hippocampus, amygdala, cerebellum, brainstem, and cerebrospinal fluid (CSF). Brain extraction was performed using the Brain Extraction Tool (BET) in the FMRIB Software Library (FSL, http://www.fmrib.ox.ac.uk/fsl in the public domain). Brain tissue segmentation was performed automatically in the MANTiS pipeline, except for the BET step. The segmentation process involved two steps. First, the initial tissue was classified using the “new segment” tool in SPM12 with the neonate probability map included in MANTiS. Second, to ensure reliable segmentation despite the presence of large ventricles or high-intensity WM, morphological watershed segmentation and filtering were carried out (Figure 1).

### DTI preprocessing

**Figure 2:**
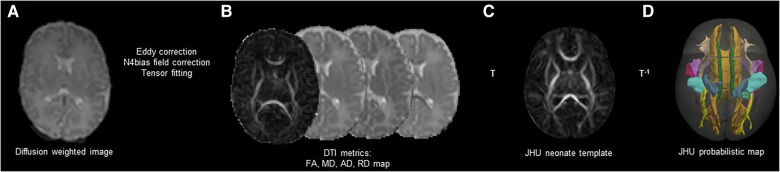
Schematic representation of DTI preprocessing. Correction of Eddy current distortions, motions artifacts, and MR susceptibility-induced field were conducted in the individual DWIs (**A**). Four scalar maps (FA, MD, AD, and RD) were calculated by voxel-wise diffusion tensor modeling (**B**). The individual DWIs were registered to the JHU-neonate white matter atlas from Johns Hopkins University under a probabilistic threshold of 0.1 (**C**). The JHU probabilistic atlas labels were registered as the individual DWIs with inverse transformation (**D**). DTI, diffusion tensor imaging; MR, magnetic resonance; DWI, diffusion weighted image; FA, fractional anisotropy; MD, mean diffusivity; AD, axial diffusivity; RD, radial diffusivity; JHU, Johns Hopkins University; T, transformation.

**Figure 3:**
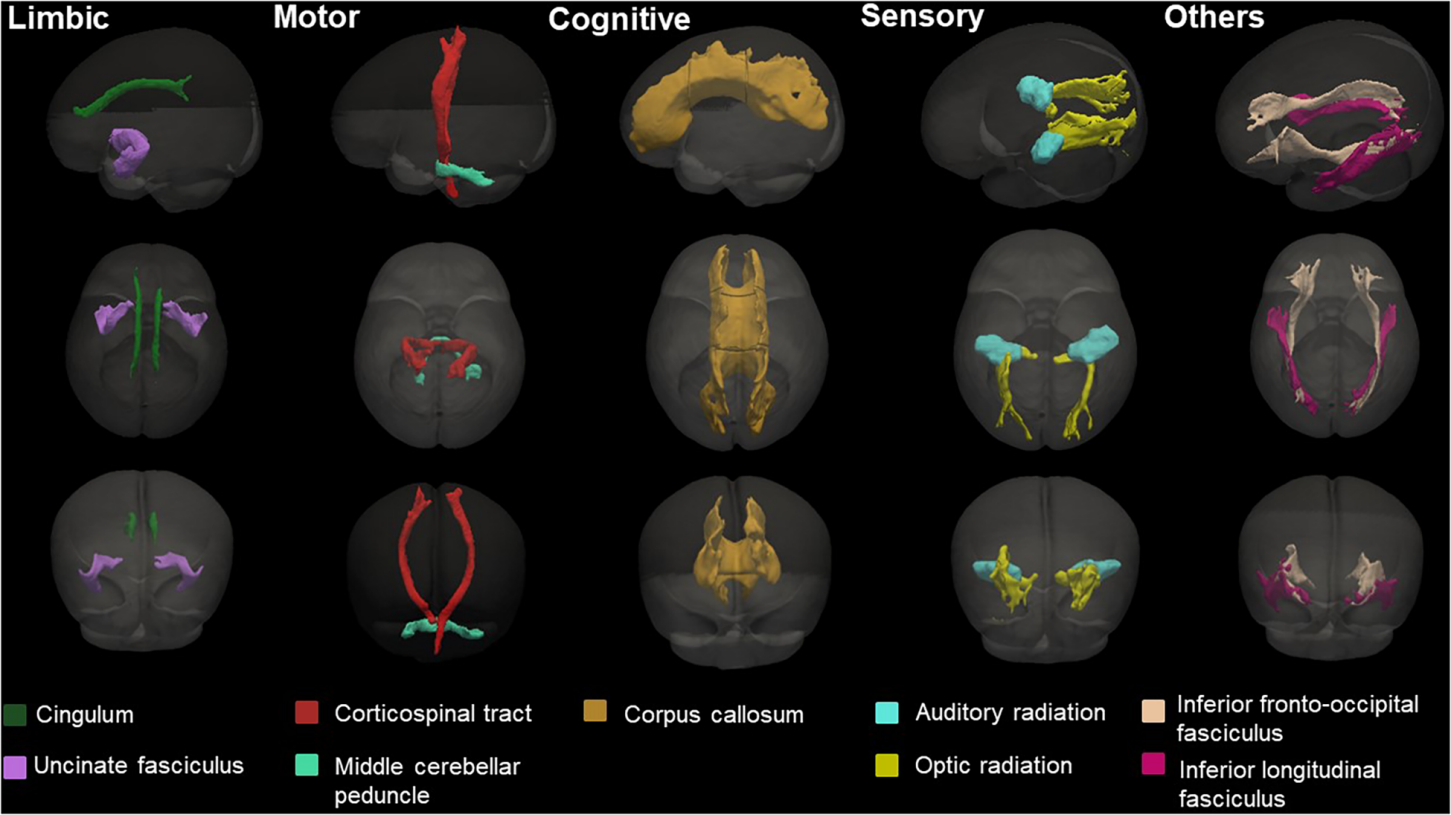
Probabilistic maps of the white matter tracts of the JHU-neonatal atlas analyzed in this study and known associated functions. The three-dimensional (3D) surface of the tracts was visualized with the brain surface of the JHU-neonate-SS template.

DTI was processed using the FSL (37). We removed the skull and non-brain tissues from the non-diffusion-weighted image (b0) and created a brain mask using BET. The MRI susceptibility-induced fields, motion artifacts, and eddy current distortions were corrected by outlier replacement using an eddy correction tool. To avoid low-frequency intensity inhomogeneity in the b0 image, we performed bias field estimation by applying N4 bias field correction to Advanced Normalization Tools (ANTs) (38, 39). Subsequently, the diffusion tensor model was reconstructed using a simple least-squares fitting of each voxel in the diffusion-weighted image. FA, mean diffusivity (MD), axial diffusivity (AD), and radial diffusivity (RD) scalar maps were extracted using tensor eigenvalues (λ1, λ2, λ3) with three major diffusion directions. Finally, using the non-linear symmetric normalization algorithm of ANTs, WM probabilistic maps (10% of threshold levels) of the Johns Hopkins University (JHU) neonatal atlas were aligned to the individual FA maps, and the mean FA, MD, AD, and RD values were calculated (40) (Figure 2). We specifically selected nine WM tracts related to the limbic, motor, cognitive, sensory, and other WM tracts for the region-of-interest analysis (Figure 3). These included the cingulum and uncinate fasciculus (UNC) for the limbic system; corticospinal tract and middle cerebellar peduncle for the motor area; the entire corpus callosum for cognitive, optic, and acoustic radiation for sensory; and the other areas of the inferior fronto-occipital fasciculus and the inferior longitudinal fasciculus. The calculation was performed by averaging the left and right values of the brain hemispheres in each area.

### Statistical analysis

Statistical analyses were performed using the SPSS software (version 27.0; SPSS, Chicago, IL, USA). Student's *t*-tests or Fisher's exact test were used to compare clinical variables, volume measures, and DTI measures between the groups. We then excluded CSF from the total brain volume (sum of eight brain regions) to calculate the intracranial volume (ICV), which was corrected by normalization with the use of ICV. “Relative to ICV” is being compared in proportion to the total volume to account for individual differences in skull size. Absolute brain volume and ICV ratio (relative to ICV) measures controlled for PMA. False discovery rate (FDR) correction was used for the multiple comparisons of the eight parts of the brain tissues. Since sex and PMA have been reported as potential confounding factors associated with differences in WM development, we compared DTI measures between the groups using an analysis of covariance corrected for PMA (41). The study population was obtained by matching GA and sex; thus, we only adjusted for PMA. For multiple comparisons, the significant *p*-value adopted was FDR-corrected for the 10 WM tracts and the four DTI parameters. The FDR-corrected *p*-value of <0.05 for brain volume and microstructures was considered significant. In addition, to investigate a linear relationship between DTI measures and other neonatal factors, we evaluated the correlations between UNC and other factors identified by ANOVA, using the Spearman rank partial correlation test. Partial correlation coefficients (*R* value) and significance levels (*p*-value) were calculated, and correlations with a *p-*value of <0.05 after adjusting for PMA were considered significant.

## Results

### Clinical characteristics

Table 1 shows the baseline demographics and clinical characteristics of the study population. In this study, 32 preterm infants were included in the analysis (16 neonates born to normal-BMI women and 16 neonates born to high-BMI women). We compared the demographic characteristics between the normal- and high-BMI mothers and their newborn offspring. The normal-BMI and high-BMI pregnant women significantly differed in mean pre-pregnancy BMI (20.56 ± 1.01 vs. 28.27 ± 1.64, respectively; *p *< 0.001) and pre-pregnancy weight (54.06 ± 3.99 vs. 72.79 ± 5.75, respectively, *p *< 0.001). Other maternal and neonatal measures were not significantly different between groups.

**Table 1:** Baseline demographic and clinical characteristics of preterm infants born from normal-BMI and high-BMI mothers included in this study.

Characteristics	All infants		
	Preterm infants of normal-BMI mothers (*n* = 16)	Preterm infants of high-BMI mothers (*n* = 16)	*p*-value
Maternal			
Age at delivery (years)	34.06 ± 4.01	34.25 ± 2.96	0.881
Pre-pregnancy BMI (kg/m^2^)	20.56 ± 1.01	28.27 ± 1.64	**<0** **.** **001**
Height (cm)	162.10 ± 5.08	160.38 ± 2.68	0.242
Pre-pregnancy weight (kg)	54.06 ± 3.99	72.79 ± 5.75	**<0** **.** **001**
Gestational weight gain (kg)	6.53 ± 3.90	6.59 ± 4.72	0.968
Cesarean section, no. (%)	13 (81.3)	15 (93.8)	0.600
Histologic chorioamniotis, no. (%)	4 (25.0)	2 (12.5)	0.654
GDM, no. (%)	1 (6.3)	5 (31.3)	0.172
Gestational hypertension, no. (%)	1 (6.3)	2 (12.5)	1.000
Neonatal			
Male sex, no. (%)	6 (37.5)	5 (31.3)	0.710
Postmenstrual age at MRI (weeks)	37.59 ± 1.10	37.38 ± 1.46	0.645
Gestational age (weeks)	28.38 ± 1.83	28.36 ± 1.62	0.968
Birth length (cm)	36.31 ± 2.79	36.88 ± 2.62	0.561
Birth weight (g)	1,130.63 ± 201.64	1,136.88 ± 194.89	0.930
Weight for length SDS	−0.12 ± 1.08	0.13 ± 0.65	0.437
Head circumference	26.44 ± 1.97	26.31 ± 3.07	0.892
BPD ≥ moderate, no. (%)	2 (12.5)	4 (25)	0.327
PDA ligation, no. (%)	2 (12.5)	3 (18.8)	1.000
Proven sepsis, no. (%)	6 (37.5)	3 (18.8)	0.433
ROP ≥ stage 3, no. (%)	2 (12.5)	0	0.484
NEC ≥ stage 2b no. (%)	0	1 (6.3)	1.000
IVH ≥ grade 2, no. (%)	0	0	1.000
Use of inotropes	1 (6.3)	0	1.000
Length of hospitalization (days)	71.25 ± 25.36	76.44 ± 24.88	0.564
Days of ventilation (days)	7.94 ± 11.26	7.00 ± 8.26	0.790
Apgar score (min)			
1	3.50 ± 1.15	3.00 ± 1.51	0.300
5	6.50 ± 0.97	6.00 ± 1.32	0.230

GDM, gestational diabetes mellitus; SDS, standard deviation score; BPD, bronchopulmonary dysplasia; PDA, patent ductus arteriosus; ROP, retinopathy of prematurity; NEC, necrotizing enterocolitis; IVH, intraventricular hemorrhage.

Data are presented as mean ± standard deviation, and significant group differences (*p* < 0.05) are highlighted in bold. Histologic chorioamnionitis was confirmed on a pathological report of the placenta. Postmenstrual age at MRI: sum of gestational age and age at MRI scan.

### Brain volume and DTI analysis

All absolute brain volumes relative to the ICV were not significantly different between the groups. However, relative to ICV after being controlled for PMA, the GM was significantly increased (44.92 ± 2.32 vs. 46.43 ± 2.13, *p *=* *0.008), and WM was decreased in the offspring of mother with high-BMI (38.94 ± 2.49 vs. 37.51 ± 2.20, *p *= 0.012) (Table 2).

**Table 2:** All subjects difference between the groups on brain volumes.

Volume measures	All infants				
	Preterm infants of normal-BMI mothers (*n* = 16)	Preterm infants of high-BMI mothers (*n* = 16)	*p*-value	Adjusted *p*^a^	FDR *p*-value^b^
Absolute volumes (mm^3^)					
Intracranial volume	310.97 ± 32.96	311.90 ± 30.24	0.934	0.698	0.785
Cortical gray matter	140.24 ± 19.01	145.23 ± 19.49	0.450	0.148	0.785
White matter	120.84 ± 12.74	116.62 ± 9.04	0.288	0.309	0.785
Cerebrospinal fluid	60.47 ± 12.92	65.39 ± 11.27	0.260	0.209	0.785
Deep gray matter	21.38 ± 2.17	21.47 ± 2.14	0.906	0.658	0.785
Hippocampus	2.44 ± 0.34	2.34 ± 0.21	0.324	0.378	0.785
Amygdala	1.44 ± 0.65	1.29 ± 0.45	0.441	0.450	0.785
Cerebellum	19.72 ± 2.18	19.84 ± 2.65	0.883	0.533	0.785
Brain stem	5.13 ± 0.89	5.12 ± 0.90	0.960	0.969	0.969
Relative to ICV (%)					
Cortical gray matter	44.92 ± 2.32	46.43 ± 2.13	0.064	0.001	**0** **.** **008**
White matter	38.94 ± 2.49	37.51 ± 2.20	0.094	0.003	**0** **.** **012**
Cerebrospinal fluid	19.55 ± 4.15	21.03 ± 3.43	0.281	0.297	0.594
Deep gray matter	6.88 ± 0.27	6.89 ± 0.37	0.926	0.923	0.925
Hippocampus	0.79 ± 0.10	0.75 ± 0.07	0.267	0.231	0.594
Amygdala	0.47 ± 0.21	0.41 ± 0.14	0.412	0.387	0.619
Cerebellum	6.35 ± 0.38	6.35 ± 0.43	0.969	0.803	0.925
Brain stem	1.66 ± 0.28	1.65 ± 0.32	0.965	0.925	0.925

BMI, body mass index; ICV, intracranial volume; FDR, false discovery rate.

Significant group differences (*p* < 0.05) are highlighted in bold.

Data are expressed as mean ± standard deviation*.*

^a^ Controlling for postmenstrual age at scan as a covariate in all preterm infants.

^b^ FDR corrected *p *< 0.05.

Figure 3 presents the representative images of the probabilistic map for each brain region, and Table 3 shows the mean DTI measures of AD for each region. The middle cerebellar peduncle and UNC were significantly lower in the offspring of high-BMI mothers. In addition, the cingulum and acoustic radiation tended to be marginally reduced. However, the statistical significance disappeared after the FDR-corrected for multiple comparisons. In total, after correction with PMA, only the UNC exhibited lower AD than the offspring of normal-BMI mothers (1.762 ± 0.101 vs. 1.690 ± 0.066, respectively; *p *=* *0.045). No significant differences were observed in the FA, MD, and RD measures between the preterm infants of normal- and high-BMI mothers (Supplementary Table S2). Maternal weight gain was not associated with the brain volume and microstructure in preterm infants (Supplementary Table S3).

**Table 3:** All subject differences between the groups on AD measures of the probabilistic map.

Pathway regions	All infants				
	Preterm infants of normal-BMI mothers (*n* = 16)	Preterm infants of high-BMI mothers (*n* = 16)	*p*-value	Adjusted *p*^a^	FDR *p*-value^b^
Corpus callosum	1.840 ± 0.076	1.824 ± 0.073	0.564	0.380	0.428
Cingulum	1.558 ± 0.074	1.513 ± 0.048	0.051	0.023	0.059
Corticospinal tract	1.857 ± 0.110	1.801 ± 0.076	0.100	0.058	0.087
Inferior fronto-occipital fasciculus	1.720 ± 0.079	1.679 ± 0.067	0.112	0.053	0.087
Inferior longitudinal fasciculus	1.762 ± 0.064	1.726 ± 0.065	0.130	0.078	0.100
Middle cerebellar peduncle	2.036 ± 0.145	1.946 ± 0.093	0.047	0.026	0.059
Optic radiation	1.787 ± 0.056	1.777 ± 0.067	0.631	0.544	0.544
Acoustic radiation	1.562 ± 0.044	1.527 ± 0.053	0.052	0.015	0.059
Uncinate fasciculus	1.762 ± 0.101	1.690 ± 0.066	0.023	0.005	**0.045**

AD, axial diffusivity; BMI, body mass index; FDR, false discovery rate.

Significant group differences (*p* < 0.05) are highlighted in bold.

Data are expressed as mean ± standard deviation.

^a^ Controlling for postmenstrual age at scan as a covariate in all preterm infants.

^b^ FDR corrected *p *< 0.05.

### Association between the UNC in AD measures and clinical factors

**Figure 4:**
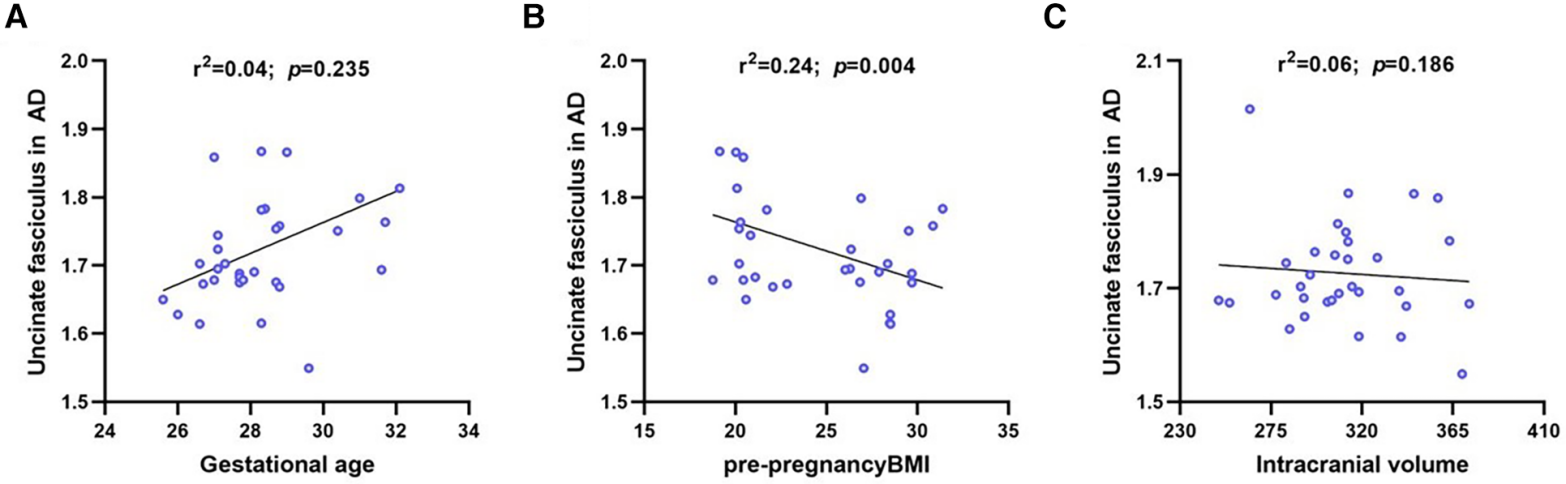
Linear regression for UNC in AD on gestational age, maternal pre-pregnancy BMI and intracranial volume. The UNC in AD was negative significantly correlated with maternal pre-pregnancy BMI, where blue circles represent each subject and black lines are linear fitting lines. UNC, uncinate fasciculus; AD, axial diffusivity; BMI, body mass index.

We analyzed the correlation between UNC in AD measures and clinical factors, such as pre-pregnancy BMI, GA, and ICV. UNC in the AD measures was positively associated with GA (*r*^2 ^= 0.04, *p *= 0.235) and negatively correlated with pre-pregnancy BMI (*r*^2 ^= 0.24, *p *= 0.004) and ICV (*r*^2 ^= 0.06, *p *= 0.186) (Figure 4).

## Discussion

This is the first study to reveal that maternal obesity affects perinatal brain development patterns at the TEA in preterm infants without apparent brain injury, suggesting that maternal obesity impacts offspring brain development. Our findings showed that preterm neonates born to high-BMI mothers had greater GM and lower WM volume than normal-BMI mothers. Furthermore, the DTI analysis showed that maternal BMI was associated with an altered microstructure of UNC on DTI analysis. These findings may establish evidence for the detrimental effects of maternal obesity on brain developmental trajectories and may mediate vulnerability to mental health in offspring during the early period in preterm infants.

Recent advances in perinatal care have substantially decreased the incidence of brain injuries such as hypoxic-ischemic encephalopathy, intraventricular hemorrhage, and periventricular leukomalacia in very preterm infants. Unfortunately, despite furtherance in perinatal care, a long-term follow-up study in children who were very preterm at birth reported the occurrence of various neurodevelopmental disorders such as cognitive and language impairments and learning disabilities (42). Considering the potential for neurodevelopmental morbidity in very preterm infants (<32 weeks of gestation), these patients were included in our follow-up programs aimed at the early detection of neurodevelopmental problems. The neural circuits of infants are shaped by a rapidly evolving series of cellular events during the third trimester of pregnancy, including synaptogenesis, neuronal migration, and myelination, which underscores the significance of this sensitive phase for infant brain plasticity. Thus, very preterm infants should be subject to strict neuromonitoring to achieve normal neurodevelopmental outcomes. In comparison to the neurodevelopmental morbidity in term-born offspring of pre-pregnancy obese mothers, those in preterm neonates have been relatively understudied. Therefore, the impact of pre-pregnancy maternal obesity on perinatal brain developmental patterns in very preterm infants (less than 32 weeks of gestation) deserves special consideration. Recently, maternal obesity has been highlighted as a risk factor for neurodevelopmental impairment in preterm infants via placental programming and perinatal inflammation (43). Moreover, maternal obesity was found to be strongly correlated with one of the risk factors for cognitive impairment and increased risk for autism in extremely preterm infants, and consistent results on adverse cognitive outcomes have also been shown in preterm infants at 2 years of age (26, 44, 45). In preterm infants, compelling evidence of a negative relationship between maternal obesity prior to pregnancy and offspring neurodevelopment appears to be confirmed by the ELGAN study, in which preterm children born to mothers with obesity were likely to have lower scores on intelligence tests at 10 years of age compared to those born to normal-BMI mothers (46). Patterns consistent with premature brain aging have been identified in the umbilical cord of fetuses exposed to maternal obesity, modifying gene expression profiles related to synaptic plasticity and synaptic growth (47, 48). Given that pre- and perinatal inflammation associated with preterm birth tends to increase sensitivity to inflammation, exposure to pre-pregnancy maternal obesity in preterm infants may increase the propensity to develop neuroinflammation during the early stages of life as an antecedent of neurodevelopmental impairment and psychiatric morbidities. By modifying the prenatal antecedents of impaired neurodevelopment, preventive approaches and targeted interventions can be optimized for extremely preterm infants.

We found significant differences in the volume measurement of both the GM and WM in preterm infants of high-BMI mothers. These findings suggest that maternal high BMI can lead to changes in neurodevelopmental trajectories. Various studies on structural abnormalities in the GM volume associated with obesity have been conducted in children and adolescents (49–51). A meta-analysis of volumetric study on GM alterations has previously reported that the volume in the inferior frontal gyrus, left middle temporal cortex, left precentral gyrus, and cerebellum is reduced in adults with obesity (52). Notably, Na et al. (20) reported that maternal pre-pregnancy obesity affects the neonatal brain cortical development in the frontal lobe; this finding suggests that the frontal lobe is selectively vulnerable to the potential impact of maternal pre-pregnancy obesity as an antecedent of neurodevelopmental impairment, showing lower cortical thickness in language and executive function-related brain regions. Higher functional connectivity within the left superior frontal gyrus in neonates is associated with maternal pre-pregnancy BMI whereas lower functional connectivity in the dorsal anterior cingulate correlates with maternal pre-pregnancy BMI (22, 24). Although it is clear that maternal obesity affects brain structure and connectivity in offspring, the effect of maternal obesity on GA at birth deserves further study. Different aspects from different modalities between structural and functional MRI and inconsistent results due to differences in study populations in terms of prematurity-related factors could represent a critical gap in our understanding of the brain connectivity in offspring affected by maternal pre-pregnancy obesity. Structural MRI methods might predominantly capture monosynaptic connections, whereas functional MRI techniques could exhibit sensitivity to polysynaptic connections (53). Study of using DTI analysis based on tract-based spatial statistics (TBSS) have shown lower FA values in widespread WM regions in newborn offspring born to mothers with obesity, indicating lower WM integrity and delayed myelination (21). In the current study, the microstructure was evaluated using the probabilistic map, which is a probabilistic tractography of the neonatal functional pathways. Both the TBSS and probabilistic map method can be sensitive in detecting the brain microstructure. However, the probabilistic map method targets the minimally myelinated superficially located WM fibers, which may result in relatively low FA values (40). On the contrary, the TBSS approach, which focuses on the WM tracts with high-FA, could have limitations in investigating these fibers.

The connection between maternal pre-pregnancy obesity and neonatal structural/functional brain development is predominantly supported by term-born subjects at birth, while the relationship between maternal pre-pregnancy obesity and the brain development of preterm infants is not well established. We showed that the WM microstructure in AD of the UNC in preterm infants was altered by maternal pre-pregnancy obesity, reflecting reduced axon density or caliber, or less coherent axonal orientation (54–56). The AD measure, the principal diffusion direction within a voxel, generally increases with brain maturation in the neonatal period, reflecting fiber coherence and the axonal state (57, 58). Lower AD in high-BMI mothers and their offspring could reflect changes in abnormal microstructural patterns due to delayed axonal development during perinatal development. The UNC is commonly considered the limbic area involved in socioemotional and cognitive problems. Furthermore, the UNC is a bidirectional pathway connecting the orbitofrontal cortex and the temporal polar cortex/Brodmann area 38, which is important for expressive language function. In terms of WM developmental patterns in the neonatal brain, the UNC seems to be very vulnerable with the longest period of development. Recent studies have shown that exposure to unpredictable maternal signals and maternal stress in infants leads to aberrant maturation of the UNC in preterm neonates (59, 60). We support that the altered trajectory of WM development in these language- and emotional function-related brain regions in early life may have important implications, leading to lower composite language scores or high risk for autism. As demonstrated in the previous study, neonates exposed to mothers with pre-pregnancy obesity are considered vulnerable to potential disturbances in the frontal lobe area, but preterm neonates born to high-BMI mothers in the current study seem to be selectively affected by other mechanisms with the alteration of the UNC WM tract. Only the study of Reynolds et al. (26) has examined the effect of maternal pre-pregnancy obesity on the neuroimaging measures of brain structure at TEA in preterm infants. Maternal pre-pregnancy obesity was not associated with neonatal brain volumetry, surfaces, and diffusion measures but was significantly associated with the risk of autism and developmental delay at 2 years of age. Contrary to our study findings, this cohort included preterm infants at TEA with brain injury and had a severe maternal pre-pregnancy BMI of obesity Class II (BMI = 35–39.9 kg/m^2^, extreme obesity). Although several studies have established an association between maternal pre-pregnancy obesity and neonatal brain developmental outcomes, additional studies to compare the different obesity classifications (e.g., severe obesity) should be considered.

The placenta is a temporary organ that connects the mother and baby during pregnancy and plays a crucial role in establishing an intrauterine environment for the fetus. A “placental programming” links maternal obesity itself to the subsequent neurodevelopmental outcomes in offspring, with lasting deleterious influence on neurodevelopment across the life span. The role of the placenta in relation to the neurodevelopment of children from mothers with obesity is well-defined in several studies, which have evaluated the role of epigenetic factors in maternal obesity-associated programming of offspring neurodevelopmental outcomes (14, 61). One of the underlying mechanisms is epigenetic modifications of the genome, such as DNA methylation, posttranslational histone modifications, and microRNA variation, without changes in the DNA sequence, which mediate fetal mal-programming in the setting of maternal obesity (62–64). Exposure to impaired placentation resulting from obesity has caused epigenetic modifications in the offspring, which may increase susceptibility to neurodevelopmental and psychiatric morbidity in offspring. The second putative mechanism involves immune and inflammatory placental changes in obese human pregnancy, which would induce placental macrophage accumulation, activation of proinflammatory cytokines, and histopathological inflammation (65, 66). Both human and animal models have demonstrated that maternal obesity induces IL-6 and TNF-α in umbilical cord blood and offspring peripheral blood (67–70). IL-6 and TNF-α are involved in the reduction of axon outgrowth within the hypothalamic circuit and sympathetic nervous system, in addition to disrupting developmental processes from the initial central nervous system formation to synaptogenesis (71). Finally, the altered plasticity of brain-derived neurotrophic factor (BDNF) is associated with maternal obesity. BDNF, one of the neurotrophic factors, plays an important role in promoting neuronal survival and regulating synaptic repair in the hippocampus (72). Animal studies have demonstrated that maternal obesity leads to deficits in spatial learning and memory in the offspring, reducing the BDNF protein levels in the hippocampus (73). Perturbations in hippocampal BDNF production may provide insights into the mechanisms underlying neurodevelopmental abnormalities in offspring exposed to maternal obesity.

The limitations of this study include BMI measurements. This study did not quantify the amount of body fat (vs. lean body mass) and identify the areas of fat deposition. Investigating maternal fat measurements and fat deposition locations in addition to maternal BMI would allow for further exploration of unresolved questions in relevant studies. Another limitation is the small sample size and collection of only MR imaging data at the neonatal age, with the absence of follow-up MR imaging. Preterm children could be followed up in a subsequent study investigating language and social–emotional outcomes at the age of 5 years. A large-scale study may allow us to confirm the local brain regions in the developing brain that are associated with maternal obesity, which could provide additional insights. Finally, measures of diffusion, especially in the right UNC region, involve the WM adjacent to the frontal lobe, the region most implicated in previous studies of maternal obesity. Although we performed several brain measurements, we did not measure surface-based measures, such as frontal thickness and volume, owing to challenges in segmentation in the newborn brain.

## Conclusion

An altered UNC microstructure, which is important for language and socioemotional function, was associated with maternal BMI in preterm infants, suggesting the use of imaging-based biomarkers at TEA for early identification and timely intervention. The influence of maternal pre-pregnancy obesity as a non-genetic factor provides clues to potential neurodevelopmental consequences in the offspring of mothers with obesity. Given that preterm birth may predispose offspring to a higher risk of neurodevelopmental delay, this study highlights the importance of detecting and preventing developmental delay in preterm infants with maternal pre-pregnancy obesity. Our results address an important basis for future follow-up studies to reveal whether the *in utero* effects of maternal obesity on offspring brain development are persistent beyond the newborn age or not.

## Data Availability

The original contributions presented in the study are included in the article/**Supplementary Material**, further inquiries can be directed to the corresponding author
