# Supplementary material for: Maternal pre-pregnancy obesity affects the uncinate fasciculus white matter tract in preterm infants
Source: Front Pediatr. 2023 Nov 15;11:1225960. doi: 10.3389/fped.2023.1225960 (PMC10684693; doi:10.3389/fped.2023.1225960)
Supplement: Supplementary file 1 [file Datasheet1.docx]

Supplementary Material

Maternal pre-pregnancy obesity affects uncinate fasciculus white matter tract in preterm infants

**Joo Young Lee^1^, Hyun Ju Lee^2,3^, Yong Hun Jang^1^, Hyuna Kim^1^, Kiho Im^4,5^,** **Seung Yang^2,6^, Jeong-Kyu Hoh^7,8^ and Ja-Hye Ahn^2,3*^**

**Affiliations**

^1^Department of Translational Medicine, Hanyang University Graduate School of Biomedical Science and Engineering, Seoul, South Korea

^2^Department of Pediatrics, Hanyang University College of Medicine, Seoul, South Korea

^3^Division of Neonatology and Development Medicine, Hanyang University Hospital, Seoul, South Korea

^4^Fetal Neonatal Neuroimaging and Developmental Science Center, Boston Children’s Hospital, Harvard Medical School, Boston, MA, USA

^5^Division of Newborn Medicine, Boston Children’s Hospital, Harvard Medical School, Boston, MA, USA^2^

^6^Department of Pediatrics, Hanyang University Hospital, Seoul, South Korea

^7^Department of Obstetrics and Gynecology, Hanyang University College of Medicine, Seoul, South Korea

^8^Department of Obstetrics and Gynecology, Hanyang University Hospital, Seoul, South Korea

***Correspondence:**

Ja Hye Ahn, MD

Tel.: 82-2-2290-8399

Fax.: 82-2-2297-2380
mdscully@gmail.com

# Supplementary Tables

**Supplementary Table 1. Seven studies using neuroimaging to analyze the brain difference in neonates born to mothers with normal-BMI and high-BMI**

|  | **Neonatal** | | | | **Maternal pre-pregnancy BMI** | | **Method** | | | **Result** | |
| --- | --- | --- | --- | --- | --- | --- | --- | --- | --- | --- | --- |
| **Study** | **Subject** | **Non-obese from mothers (N)** | **Obese from mothers (N)** | **Age at MRI** | **Measured criteria** | **Definition of exposure (Obese)** | **Neuroimaging technique** | **Analytical method** | **Confounding factors** | **Significant changes in the regions** | **Group difference** |
| Na et al., 2021 (1) | Full-term offspring | 28 | 16 | within 2 weeks | Measured at ~ 12 weeks of gestation | BMI ≥ 30 | Structural MRI | Surface analysis | Sex, Race, PMA, Maternal age/IQ/education | Cortical thickness in Lt.frontal lobe (pars opercularis gyrus, pars triangularis gyrus, rostral middle frontal gyrus) | High-BMI < Normal-BMI |
| Ou et al., 2015 (2) | Full-term offspring | 17 | 11 | within 2 weeks | Measured at ~ 10 weeks of gestation | BMI ≥ 30 | DTI | TBSS | Sex, Birth weight/length/HC, Neonatal diet, PMA, GWG | FA value in multiple white matter regions (association, projection, callosal and lymbic fibers) | High-BMI < Normal-BMI |
| Li et al., 2016 (3) | Full-term offspring | 18 | 16 | within 2 weeks | Self-reported | BMI ≥ 30 | rs-fMRI | Functional connectivity | Sex, PMA, Birth weight/length/HC, Neonatal diet, Maternal IQ, GWG | Connectivity in both dorsal anterior cingulate | High-BMI < Normal-BMI |
| Salzwedel et al., 2018 (4) | Full-term offspring | 23 | 15 | within 2 weeks | Self-reported | BMI > 25 | rs-fMRI | Functional connectivity | NA | Hypo connectivity in between Rt.caudate and Rt.inferior orbital frontal gyrus | High-BMI < Normal-BMI |
| Rajasilta et al., 2021 (5) | Full-term offspring | 10 | 11 | within 2 weeks | Self-reported | BMI ≥ 25 | rs-fMRI | Functional connectivity | Sex, GA, PMA | Connectivity in Lt.Superior frontal gyrus | High-BMI > Normal-BMI |
| Spann et al., 2020 (6) | Full-term offspring | 17 | 21 | within 3 weeks | Self-reported | BMI ≥ 25 | rs-fMRI | Functional connectivity | Sex, PMA, Scanner type | Connectivity in Lt.thalamus | High-BMI > Normal-BMI |
| Reynolds et al., 2014 (7) | Preterm offspring* | 38 | 24 | TEA | Measured at ~ 12 weeks of gestation | BMI ≥ 30 | Structural MRI & DTI | Brain volumetry, Surface analysis, ROI based DTI-metric analysis | Sex, GA, PMA | No significant change | NA |

*Preterm infants who were born ≤ 30 weeks gestation. Abbreviations: BMI, body mass index; MRI, magnetic resonance imaging; PMA, postmenstrual age; IQ, intelligence quotient; Lt, left; Rt, right; DTI, diffusion tensor imaging; TBSS, tract-based spatial statistics; HC, head circumference; GWG, gestational weight gain; FA, fractional anisotropy; rs-fMRI, resting-state functional magnetic resonance imaging; TEA; term-equivalent age; ROI, region of interest; NA, not available

**Supplementary Table 2. All subjects difference between the groups on FA, MD and RD measures of probabilistic map**

| **Pathway**  **regions** | **FA** | | | **MD** | | | **RD** | | |
| --- | --- | --- | --- | --- | --- | --- | --- | --- | --- |
|  | **Preterm infants of mothers with Normal-BMI** | **Preterm infants of mothers with High -BMI** | ***Adjusted***  ***P-value**** | **Preterm infants of mothers with Normal-BMI** | **Preterm infants of mothers with High -BMI** | ***Adjusted***  ***P-value**** | **Preterm infants of mothers with Normal-BMI** | **Preterm infants of mothers with High -BMI** | ***Adjusted***  ***P-value**** |
| CC | 0.170±0.017 | 0.169±0.016 | 0.984 | 1.572±0.071 | 1.559±0.076 | 0.403 | 1.438±0.072 | 1.427±0.080 | 0.442 |
| CG | 0.151±0.013 | 0.153±0.012 | 0.788 | 1.352±0.069 | 1.309±0.052 | 0.075 | 1.249±0.069 | 1.208±0.054 | 0.108 |
| CST | 0.243±0.023 | 0.252±0.025 | 0.444 | 1.491±0.109 | 1.434±0.083 | 0.090 | 1.308±0.112 | 1.251±0.089 | 0.114 |
| IFO | 0.166±0.019 | 0.168±0.016 | 0.788 | 1.474±0.764 | 1.436±0.065 | 0.090 | 1.346±0.075 | 1.318±0.065 | 0.197 |
| ILF | 0.168±0.015 | 0.170±0.014 | 0.788 | 1.507±0.062 | 1.472±0.065 | 0.090 | 1.380±0.064 | 1.345±0.067 | 0.114 |
| MCP | 0.198±0.026 | 0.195±0.023 | 0.899 | 1.709±0.125 | 1.638±0.092 | 0.090 | 1.546±0.122 | 1.484±0.095 | 0.114 |
| OR | 0.209±0.019 | 0.219±0.019 | 0.365 | 1.474±0.063 | 1.454±0.073 | 0.342 | 1.317±0.069 | 1.293±0.078 | 0.263 |
| AR | 0.198±0.015 | 0.205±0.015 | 0.365 | 1.295±0.046 | 1.256±0.057 | 0.063 | 1.161±0.050 | 1.121±0.059 | 0.099 |
| UNC | 0.167±0.025 | 0.161±0.015 | 0.788 | 1.509±0.083 | 1.454±0.069 | 0.063 | 1.383±0.081 | 1.336±0.072 | 0.108 |

Data are expressed as mean ± standard deviation. *Controlling for age at scan as covariate in all preterm infants and statistical significance is p < 0.05 after FDR-corrected. Abbreviations: FA, fractional anisotropy; MD, mean diffusivity; RD, radial diffusivity; BMI, body mass index; CC, corpus callosum; CG, cingulum; CST, corticospinal tract; IFO, inferior fronto-occipital fasciculus; ILF, inferior longitudinal fasciculus; MCP, middle cerebellar peduncle; OR, optic radiation; AR, acoustic radiation; UNC, uncinate fasciculus

**Supplementary Table 3. Association between weekly gestational weight gain and brain measurements of preterm infants**

| **Pathway regions** | **Weekly gestational weight gain** | | | | |
| --- | --- | --- | --- | --- | --- |
|  | **Unadjusted analysis**  **Coef (95%CI)** | ***P-value*** | **Adjusted analysis*****  **Coef (95%CI)** | ***Adjusted-P*** | ***FDR***  ***P-value*** |
| **Volume measures** |  |  |  |  |  |
| **Cortical gray matter** | 23.7 (-14.90,62.20) | 0.220 | -0.457 (-14.56,13.64) | 0.947 | 0.980 |
| **White matter** | 17.48 (-12.80,47.75) | 0.247 | -0.192 (-15.61,15.22) | 0.980 | 0.980 |
| **Cerebrospinal fluid** | -1.23 (-34.49,32.02) | 0.940 | -6.201 (-40.28,27.88) | 0.712 | 0.980 |
| **Deep gray matter** | 2.417 (-2.46,7.29) | 0.319 | -0.334 (-3.01,2.40) | 0.804 | 0.980 |
| **Hippocampus** | -0.103 (-0.87,0.66) | 0.785 | -0.344 (-1.05,0.36) | 0.328 | 0.980 |
| **Amygdala** | 0.197 (-1.36,1.75) | 0.797 | 0.074 (-1.55,1.70) | 0.926 | 0.980 |
| **Cerebellum** | 4.249 (-0.38,8.88) | 0.071 | 1.865 (-1.23,4.96) | 0.228 | 0.980 |
| **Brain stem** | -0.359 (-2.78,2.06) | 0.764 | -0.612 (-3.12,1.90) | 0.621 | 0.980 |
| **Pathway regions_FA** |  |  |  |  |  |
| **Corpus callosum** | -0.026 (-0.07,0.02) | 0.223 | -0.021 (-0.06,0.02) | 0.312 | 0.562 |
| **Cingulum** | -0.023 (-0.06,0.01) | 0.162 | -0.020 (-0.05,0.01) | 0.220 | 0.562 |
| **Corticospinal tract** | -0.017 (-0.08,0.05) | 0.594 | -0.002 (-0.06,0.05) | 0.938 | 0.938 |
| **Inferior fronto-occipital fasciculus** | -0.030 (-0.08,0.02) | 0.191 | -0.025 (-0.07,0.02) | 0.264 | 0.562 |
| **Inferior longitudinal fasciculus** | -0.014 (-0.05,0.03) | 0.489 | -0.010 (-0.05,0.03) | 0.616 | 0.792 |
| **Middle cerebellar peduncle** | 0.019 (-0.05,0.09) | 0.552 | 0.027 (-0.04,0.09) | 0.397 | 0.596 |
| **Optic radiation** | -0.040 (-0.09,0.01) | 0.120 | -0.030 (-0.08,0.02) | 0.201 | 0.562 |
| **Acoustic radiation** | -0.028 (-0.07,0.01) | 0.173 | -0.021 (-0.06,0.02) | 0.272 | 0.562 |
| **Uncinate fasciculus** | -0.011 (-0.07,0.04) | 0.692 | -0.009 (-0.07,0.05) | 0.744 | 0.837 |
| **Pathway regions_MD** |  |  |  |  |  |
| **Corpus callosum** | 0.193 (-0.01,0.38) | 0.041 | 0.154 (-0.01,0.32) | 0.067 | 0.153 |
| **Cingulum** | 0.212 (0.06,0.37) | 0.009 | 0.189 (0.04,0.34) | 0.015 | 0.135 |
| **Corticospinal tract** | 0.178 (-0.09,0.44) | 0.177 | 0.135 (-0.12,0.39) | 0.279 | 0.359 |
| **Inferior fronto-occipital fasciculus** | 0.192 (0.01,0.38) | 0.041 | 0.161 (-0.01,0.34) | 0.068 | 0.153 |
| **Inferior longitudinal fasciculus** | 0.152 (-0.02,0.32) | 0.072 | 0.128 (-0.03,0.29) | 0.117 | 0.211 |
| **Middle cerebellar peduncle** | 0.112 (-0.20,0.42) | 0.463 | 0.070 (-0.23,0.37) | 0.638 | 0.638 |
| **Optic radiation** | 0.091 (-0.09,0.27) | 0.313 | 0.064 (-0.11,0.24) | 0.460 | 0.518 |
| **Acoustic radiation** | 0.155 (0.02,0.29) | 0.027 | 0.128 (0.00,0.25) | 0.045 | 0.153 |
| **Uncinate fasciculus** | 0.163 (-0.05,0.37) | 0.124 | 0.124 (-0.07,0.32) | 0.204 | 0.306 |
| **Pathway regions_AD** |  |  |  |  |  |
| **Corpus callosum** | 0.181 (-0.01,0.37) | 0.062 | 0.145 (-0.03,0.32) | 0.103 | 0.200 |
| **Cingulum** | 0.211 (0.05,0.37) | 0.011 | 0.189 (0.03,0.35) | 0.019 | 0.171 |
| **Corticospinal tract** | 0.186 (-0.07,0.44) | 0.146 | 0.154 (-0.10,0.41) | 0.221 | 0.662 |
| **Inferior fronto-occipital fasciculus** | 0.180 (-0.01,0.36) | 0.056 | 0.150 (-0.03,0.33) | 0.092 | 0.200 |
| **Inferior longitudinal fasciculus** | 0.155 (-0.01,0.32) | 0.070 | 0.133 (-0.03,0.30) | 0.111 | 0.200 |
| **Middle cerebellar peduncle** | 0.154 (-0.19,0.50) | 0.369 | 0.114 (-0.23,0.46) | 0.501 | 0.564 |
| **Optic radiation** | 0.050 (-0.12,0.22) | 0.540 | 0.033 (-0.13,0.20) | 0.683 | 0.683 |
| **Acoustic radiation** | 0.139 (0.01,0.27) | 0.036 | 0.115 (-0.01,0.24) | 0.059 | 0.200 |
| **Uncinate fasciculus** | 0.170 (-0.07,0.41) | 0.160 | 0.127 (-0.10,0.35) | 0.259 | 0.333 |
| **Pathway regions_RD** |  |  |  |  |  |
| **Corpus callosum** | 0.198 (0.01,0.39) | 0.041 | 0.157 (-0.01,0.33) | 0.067 | 0.151 |
| **Cingulum** | 0.213 (0.06,0.37) | 0.009 | 0.189 (0.04,0.34) | 0.016 | 0.141 |
| **Corticospinal tract** | 0.174 (-0.10,0.45) | 0.206 | 0.126 (-0.13,0.39) | 0.328 | 0.422 |
| **Inferior fronto-occipital fasciculus** | 0.204 (0.03,0.38) | 0.025 | 0.176 (0.01,0.35) | 0.041 | 0.141 |
| **Inferior longitudinal fasciculus** | 0.151 (-0.02,0.32) | 0.084 | 0.125 (-0.04,0.29) | 0.136 | 0.245 |
| **Middle cerebellar peduncle** | 0.090 (-0.21,0.39) | 0.548 | 0.047 (-0.25,0.34) | 0.748 | 0.748 |
| **Optic radiation** | 0.112 (-0.08,0.31) | 0.252 | 0.080 (-0.11,0.27) | 0.389 | 0.438 |
| **Acoustic radiation** | 0.162 (0.02,0.31) | 0.029 | 0.135 (0.00,0.27) | 0.047 | 0.141 |
| **Uncinate fasciculus** | 0.159 (-0.05,0.37) | 0.126 | 0.123 (-0.07,0.32) | 0.207 | 0.311 |

Data are expressed as mean ± standard deviation. A statistical significance is p < 0.05 after FDR-corrected. *Controlling for postmenstrual age at scan and intracranial volume as covariate in volume measures and controlling for postmenstrual age at scan as covariate in pathway regions. Abbreviations: CI, confidence interval; FDR, false discovery rate; FA, fractional anisotropy; MD, mean diffusivity; AD, axial diffusivity; RD, radial diffusivity

# References

1. Na X, Phelan NE, Tadros MR, Wu Z, Andres A, Badger TM, et al. Maternal Obesity During Pregnancy Is Associated with Lower Cortical Thickness in the Neonate Brain. *AJNR Am J Neuroradiol* (2021) 42(12):2238-44. Epub 20211007. doi: 10.3174/ajnr.A7316.

2. Ou X, Thakali KM, Shankar K, Andres A, Badger TM. Maternal Adiposity Negatively Influences Infant Brain White Matter Development. *Obesity (Silver Spring)* (2015) 23(5):1047-54. doi: 10.1002/oby.21055.

3. Li X, Andres A, Shankar K, Pivik RT, Glasier CM, Ramakrishnaiah RH, et al. Differences in Brain Functional Connectivity at Resting State in Neonates Born to Healthy Obese or Normal-Weight Mothers. *Int J Obes (Lond)* (2016) 40(12):1931-4. Epub 20160928. doi: 10.1038/ijo.2016.166.

4. Salzwedel AP, Gao W, Andres A, Badger TM, Glasier CM, Ramakrishnaiah RH, et al. Maternal Adiposity Influences Neonatal Brain Functional Connectivity. *Front Hum Neurosci* (2018) 12:514. Epub 20190104. doi: 10.3389/fnhum.2018.00514.

5. Rajasilta O, Hakkinen S, Bjornsdotter M, Scheinin NM, Lehtola SJ, Saunavaara J, et al. Maternal Pre-Pregnancy Bmi Associates with Neonate Local and Distal Functional Connectivity of the Left Superior Frontal Gyrus. *Sci Rep* (2021) 11(1):19182. Epub 20210928. doi: 10.1038/s41598-021-98574-9.

6. Spann MN, Scheinost D, Feng T, Barbato K, Lee S, Monk C, et al. Association of Maternal Prepregnancy Body Mass Index with Fetal Growth and Neonatal Thalamic Brain Connectivity among Adolescent and Young Women. *JAMA Netw Open* (2020) 3(11):e2024661. Epub 20201102. doi: 10.1001/jamanetworkopen.2020.24661.

7. Reynolds LC, Inder TE, Neil JJ, Pineda RG, Rogers CE. Maternal Obesity and Increased Risk for Autism and Developmental Delay among Very Preterm Infants. *J Perinatol* (2014) 34(9):688-92. Epub 20140508. doi: 10.1038/jp.2014.80.
